# Noninvasive ventilation in the emergency setting: predictors of failure and long-term mortality and impact on health-related quality of life

**DOI:** 10.1186/2197-425X-3-S1-A175

**Published:** 2015-10-01

**Authors:** M Vilaça, C Dias, I Aragão, G Campello

**Affiliations:** Medicine Integrated Master (MIM), Instituto de Ciência Biomédicas Abel Salazar, Porto, Portugal; Faculdade de Medicina da Universidade do Porto, Information Sciences and Decision on Health Department (CIDES), Porto, Portugal; Intensive Care Unit (UCIP) - Centro Hospitalar do Porto, Porto, Portugal

## Introduction

Noninvasive ventilation (NIV) has been used increasingly outside intensive care units. Predictors of success and failure have been poorly explored in these settings. To our knowledge no study has evaluated the impact of success or failure of NIV on mortality and health-related quality of life (HRQOL) in an emergency setting.

## Objectives

To evaluate independent risk factors associated with NIV failure and mortality in patients admitted to the emergency room and its impact on HRQOL.

## Methods

Prospective cohort study in all patients admitted to the emergency room of a tertiary care, university-affiliated, 600-bed hospital between January and December 2014, who received NIV for acute or acute-on-chronic respiratory failure with no withhold treatment decision. NIV failure was defined as the need for endotracheal intubation. Association of variables with NIV failure was studied through logistic regression models. HRQOL was evaluated using SF-12. Long-term outcome was evaluated at 90 days after hospital discharge by a telephone interview.

## Results

During the study period, 1727 patients were admitted to the emergency room, of which 243 were included in the study. Of those 70 (29%) had a “do not intubate” order and were excluded. NIV failed in 40 (24%). Variables associated with NIV failure were: young age [adjusted OR (95% CI) = 0,97(0,94-0,99)], pneumonia [adjusted OR (95% CI) = 3,86(1,60-9,41)], sepsis at admission [adjusted OR (95% CI) = 3,34(1,08-10,30)] and lower values of bicarbonates 1 hour after NIV [adjusted OR (95% CI) = 0,92(0,87-0,98)]. Acute cardiogenic pulmonary edema was significantly more frequent in the success group (52% vs 24%, p = 0.002). Immunosuppression (6% vs 22%, p = 0.006) and active cancer (11% vs 24%, p = 0.026) were more frequent in the failure group. The hospital mortality rate was significantly higher (54%) in the group of patients with failed NIV than in the success group (10%), (p < 0,001). Independent risk factors for hospital mortality were: NIV failure [adjusted OR (95% CI) = 14,31(5,22-39,22)], pneumonia at admission [adjusted OR (95% CI) = 5,02(2,06-12,27)], neuromuscular diseases [adjusted OR (95% CI) = 8,64(1,74-43,00)] and lower values of lactates after 1 hour of NVI [adjusted OR (95% CI) = 1,40(1,08-1,82)]. No difference was found in HRQOL between the two groups at admission (baseline) and 90 days after hospital discharge. No significant decline on HRQOL was observed in the two groups.

## Conclusions

Pneumonia, sepsis and lower values of bicarbonates 1 hour after NIV were independent risk factors for NIV failure. NIV failure, pneumonia at admission, neuromuscular diseases and lower values of lactates 1 hour after NIV were independent risk factors for higher hospital mortality. No significant impact on HRQOL was observed in both groups at admission or in the long-term follow-up.

**Figure 1:**
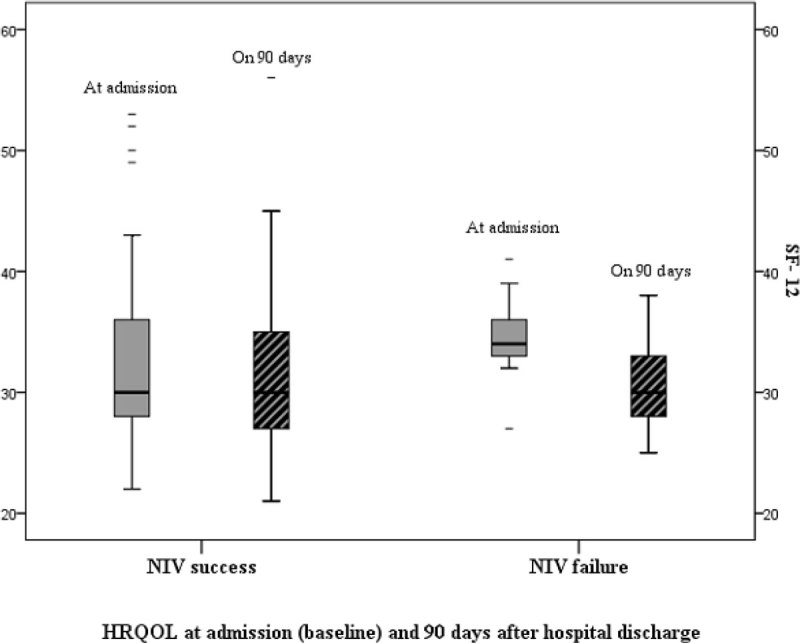
**HRQOL at admition and on 90 days after discharge.**
